# Tumor-Experienced Human NK Cells Express High Levels of PD-L1 and Inhibit CD8^+^ T Cell Proliferation

**DOI:** 10.3389/fimmu.2021.745939

**Published:** 2021-09-20

**Authors:** Jessica M. Sierra, Florencia Secchiari, Sol Y. Nuñez, Ximena L. Raffo Iraolagoitia, Andrea Ziblat, Adrián D. Friedrich, María V. Regge, M. Cecilia Santilli, Nicolás I. Torres, Mariana Gantov, Aldana Trotta, Carlos Ameri, Gonzalo Vitagliano, Hernando Ríos Pita, Luis Rico, Agustín Rovegno, Nicolás Richards, Carolina I. Domaica, Norberto W. Zwirner, Mercedes B. Fuertes

**Affiliations:** ^1^Laboratorio de Fisiopatología de la Inmunidad Innata, Instituto de Biología y Medicina Experimental (IBYME), Consejo Nacional de Investigaciones Científicas y Técnicas (CONICET), Buenos Aires, Argentina; ^2^Universidad de Buenos Aires, Facultad de Farmacia y Bioquímica, Cátedra de Inmunología, Buenos Aires, Argentina; ^3^Hospital Alemán, Buenos Aires, Argentina; ^4^Centro de Educación Médica e Investigaciones Clínicas “Norberto Quirno” (CEMIC), Buenos Aires, Argentina; ^5^Departamento de Química Biológica, Facultad de Ciencias Exactas y Naturales, Universidad de Buenos Aires, Buenos Aires, Argentina

**Keywords:** NK cells, PD-L1, CD8^+^ T cells, regulation, renal cell carcinoma

## Abstract

Natural Killer (NK) cells play a key role in cancer immunosurveillance. However, NK cells from cancer patients display an altered phenotype and impaired effector functions. In addition, evidence of a regulatory role for NK cells is emerging in diverse models of viral infection, transplantation, and autoimmunity. Here, we analyzed clear cell renal cell carcinoma (ccRCC) datasets from The Cancer Genome Atlas (TCGA) and observed that a higher expression of NK cell signature genes is associated with reduced survival. Analysis of fresh tumor samples from ccRCC patients unraveled the presence of a high frequency of tumor-infiltrating PD-L1^+^ NK cells, suggesting that these NK cells might exhibit immunoregulatory functions. *In vitro*, PD-L1 expression was induced on NK cells from healthy donors (HD) upon direct tumor cell recognition through NKG2D and was further up-regulated by monocyte-derived IL-18. Moreover, *in vitro* generated PD-L1^hi^ NK cells displayed an activated phenotype and enhanced effector functions compared to PD-L1^-^ NK cells, but simultaneously, they directly inhibited CD8^+^ T cell proliferation in a PD-L1-dependent manner. Our results suggest that tumors might drive the development of PD-L1-expressing NK cells that acquire immunoregulatory functions in humans. Hence, rational manipulation of these regulatory cells emerges as a possibility that may lead to improved anti-tumor immunity in cancer patients.

## Introduction

Natural killer (NK) cells represent the cytotoxic lineage of innate lymphoid cells (ILC) (1) and play a pivotal role as effector cells against tumors and virus-infected cells. NK cell activation, which is controlled by the balance of signals elicited by a plethora of inhibitory and activating receptors, results in a cytotoxic response and the production of inflammatory cytokines, such as interferon-γ (IFN-γ) and tumor necrosis factor (TNF) (2, 3). Although NK cells are critical for the elimination of nascent tumor cells (4, 5), during the equilibrium phase (dormancy) between the tumor and the immune system NK cells seem to be dispensable (6). Moreover, once tumors escape from the immune system through a broad spectrum of immunosuppressive strategies that result in clinically apparent malignancies (7), tumor-infiltrating NK cells (TINK) become dysfunctional and display phenotypic alterations (8–13).

Recent observations demonstrated a regulatory role for NK cells in diverse models of viral infection (14–17), transplantation (18) and autoimmunity (19). For example, NK cells can limit T cell responses through multiple mechanisms, including NK cell-mediated killing of activated CD8^+^ T cells (16, 17, 20–23), CD4^+^ T cells (20, 21, 24, 25) and dendritic cells (14, 26, 27). In the context of chronic viral infections and other immunopathological conditions, such regulatory activity of NK cells may contribute to T cell homeostasis. Accordingly, some patients with autoimmune disorders display reduced NK cell numbers with impaired cytotoxicity (28). However, evidence about a regulatory function of NK cells during tumor growth remains poorly explored (29). In this context, we have recently identified a subset of Programmed Death-Ligand 1 (PD-L1)-expressing NK cells, that arise in tumor bearing mice which can inhibit tumor-specific CD8^+^ T cell priming (30).

PD-L1 is a suppressive molecule expressed on diverse hematopoietic and nonhematopoietic cells that contributes to maintain T cell homeostasis (31). However, PD-L1 is also up-regulated on tumor cells which contributes to the evasion of the immune response (31). Its receptor, PD-1, is up-regulated on activated T cells, and functions as a co-inhibitory molecule during T cell–mediated immunity (32). Targeted blockade of PD-1/PD-L1 using specific monoclonal antibodies (mAb) can restore T cell function and enhance antitumor immunity, promoting clinically unparalleled responses in different types of tumors (33, 34). Although tumor expression of PD-L1 seems to be the main driver for immune suppression, several studies showed that PD-L1 expressed by non-tumor cells including myeloid-derived cells, regulatory T cells and endothelial cells also mediate T cell disfunction (35–39). However, the expression of PD-L1 on human NK cells, its regulation and function remain ill-defined.

Here we show that the presence of a high expression of NK cell-associated signature genes in clear cell renal cell carcinoma (ccRCC) datasets -which account for 75% off all RCC (40)- from The Cancer Genome Atlas (TCGA) is associated with a worsened outcome. Also, ccRCC tumor samples, exhibited a high frequency of PD-L1^+^ TINK. PD-L1 expression was induced on NK cells after direct interaction with tumor cells and enhanced by monocyte-derived IL-18. Also, although PD-L1^+^ NK cells retained their effector functions, they inhibited CD8 T cell proliferation through PD-L1. Our results indicate that tumors drive the expansion of PD-L1-expressing NK cells with immunoregulatory functions. Accordingly, targeting of regulatory NK cells emerges as a possibility that may lead to the reinvigoration of anti-tumor immunity.

## Material and Methods

### Bioinformatic Analysis

The online tool GEPIA2 [Gene Expression Profiling Interactive Analysis, http://gepia2.cancer-pku.cn/#survival (41)] was used to analyze RNA-Seq data from TCGA. Kidney Renal Clear Cell Carcinoma (KIRC, n=516) dataset was used to perform a Kaplan-Meier analysis of the effect of NK cell infiltration on disease-free survival and overall survival. NK cell infiltration was assesses based on expression of a multi-gene signature that includes 10 NK cell-associated genes (NCR1, XCL1, XCL2, NCAM1, NCR3, IL18RAP, KIR2DL4, KLRC3, KLRD1 and NCR2; 42–45). For the analysis, patients were separated in two groups: those with a high NK cell infiltration (presence of high abundance of this NK cell signature, top quartile) and those with a low NK cell infiltration (presence of low abundance of this NK cell signature, bottom quartile). Differences between groups were analyzed with log-rank (Mantel-Cox) test and the hazard ratio (HR) was estimated using a Cox proportional hazards model in GEPIA2.

### Human Samples

Blood and tumor biopsies were obtained from treatment-naïve ccRCC patients undergoing partial or radical nephrectomy in two urology services (CEMIC and Hospital Alemán, Buenos Aires), at the time of surgery. Buffy coats from healthy donors (HD) were provided by the Hospital Churruca-Visca (Buenos Aires). The characteristics of the patients are listed in **Supplementary Table 1**. This study was conducted according to the guidelines of the Declaration of Helsinki and approved by the Institutional Ethics Committee of IBYME (protocol CE003-03/2014, date of approval: March 20, 2014). Also written informed consent of participating subjects was obtained.

### Cells and Cultures

The NK cell-sensitive K562 cell line and the human ccRCC cell line 786-O were obtained from ATCC. The RCC cell line SN12c was kindly provided by Dr. Gabriel Fiszman (Instituto de Oncología Angel H. Roffo, Buenos Aires). Cells were cultured in RPMI 1640 (Thermo-Fischer) supplemented with 10% inactivated FBS (Thermo-Fischer), sodium pyruvate (Fluka), glutamine (Sigma-Aldrich) and antibiotics (Sigma-Aldrich), and kept at 37°C in 5% CO_2_.

### Cell Separation

Peripheral blood mononuclear cells (PBMC) were obtained by Ficoll-Paque™ Plus (GE Life Sciences) centrifugation of the blood samples. Monocytes (CD14^+^ cells) were isolated by MACS (Miltenyi); NK cells and T cells were isolated using RosetteSep (StemCell) and Ficoll-Paque™ Plus centrifugation. Purity of isolated cells was always above 90%, as assessed by flow cytometry as CD14^+^ cells (monocytes), CD3^-^CD56^+^ cells (NK cells) or CD3^+^ cells (T cells).

### Flow Cytometry and Cell Sorting

Non-specific staining was blocked with 10% normal mouse serum. Cells were labeled according to the experiment with the following Ab: APC anti-CD56 (clone N901) from Beckman Coulter; PE and FITC anti-CD3 (clone UCHT1), FITC anti-CD14 (clone M5E2), PE/Cy7 anti-PD-L1 (clone 29E.2A3), PE anti-CD25 (clone BC96), PE anti-IL-18Rα (clone H44), FITC anti-CD69 (clone FN50), PE anti-IFN-γ (clone 4S.B3), Brilliant Violet 421 anti-CD107a (clone H4A3), PE anti-FasL (clone NOK-1), PerCP/Cy5-5 anti-CD34 (clone 581), APC/Cy7 anti-CD45 (clone H130) from BioLegend; AlexaFluor 488 anti-TRAIL (clone 75402) from R&D Systems; APC and violetFluor 450 anti-CD3 (clone UCHT1) and PE/Cy7 anti-CD8 (clone RPA-T8) from Tonbo Biosciences. Cell viability was determined with ZombieAqua from BioLegend. For cell sorting, PBMC were cultured 48 h alone or with K562 cells, stained with anti-CD3 and anti-CD56 mAb, and thereafter NK cells (CD3^-^CD56^+^ cells) were sorted in a FACSAria II-plus cell sorter (BD Bioscience) with a purity of 95–98%. For TINK staining, single cell suspensions from freshly dissected tumors were prepared by mechanical dissociation and filtration through nylon mesh. Cell suspensions were exhaustively washed and stained for CD34, CD45, CD3, CD56, PD-L1 and ZombieAqua. PD-L1 expression was analyzed on live NK cells (ZombieAqua^low^CD45^+^CD34^-^CD3^-^CD56^+^ cells) following the gating strategy depicted in **Supplementary Figure 1**. Fluorescence Minus One (FMO) was used to establish the gate for the PD-L1^-^ and PD-L1^+^ populations. Expression of IFN-γ was analyzed by intracellular flow cytometry using FOXP3 Fix/Perm buffer and FOXP3 Perm buffer (BioLegend). For assessment of IFN-γ production and degranulation (CD107a), cells were cultured in the presence of Monensin and Brefeldin A (BioLegend) during the last 5 h of culture and analyzed by flow cytometry. Samples were acquired in a FACSCanto II-plus flow cytometer (BD Biosciences), a MACSQuant10 or a MACSQuant16 Analyzer (Miltenyi Biotec) and data analysis was conducted with FlowJo software (BD Biosciences). Results were expressed as geometric mean fluorescence intensity (MFI) or as percentage of positive cells.

### *In Vitro* PD-L1 Expression

5x10^5^ PBMC or 1x10^5^ NK cells isolated from HD were cultured in the absence (medium) or in the presence of 3x10^5^ tumor cells for 48 h, stained for CD3, CD56 and PD-L1, and analyzed by flow cytometry following the gating strategy depicted in **Supplementary Figure 2**. For blocking experiments, anti-IL-12 p70 (10 μg/ml, clone QS-12p70, Abcam), anti-IL-15 (10 μg/ml, clone ct2nu, eBioscience), anti-IL-18 (10 μg/ml, clone 125-2H, MBL International), anti-IFN-γ (10 μg/ml, clone NIB42, BioLegend), anti-IFN-γR (10 μg/ml, clone GIR-208, BioLegend) or anti-NKG2D (20 μg/ml, clone 1D11, BioLegend) mAb, were added at the beginning of the cocultures. In other experiments, recombinant IFN-γ (200 ng/ml, Peprotech) and recombinant IL-18 (10 ng/ml, MBL International) were used to stimulate cells. In some experiments, 1x10^5^ autologous monocytes were added to NK cell cultures.

### ELISA

Detection of biologically active IL-18 in culture supernatants was performed using the anti-human IL-18 matched pair of mAb clone 125-2H and biotinylated clone 159-12B (MBL International), and HRP-labeled streptavidin (BioLegend).

### T-Cell Proliferation Assay

CFSE-labeled T cells (5 x10^4^ cells/well) were stimulated with plate-bound anti-CD3 (clone OKT3, 1.5 μg/mL, BioLegend) and soluble anti-CD28 (clone CD28.2, 1.5 μg/mL, BioLegend) mAb in the absence or in the presence of cell sorted autologous control (*NK_control_*) or tumor-experienced NK cells (*NK_te_*) at different ratios, in the absence or in the presence of a blocking anti-PD-L1 mAb (20 μg/mL, clone 29E.2A3, BioLegend). After 5 days, cells were harvested, stained for CD3, CD56, CD8, and ZombieAqua viability dye, and CFSE dilution was analyzed by flow cytometry. The proliferation was normalized to the proliferation of stimulated T cells without NK cells for each donor. Relative proliferation was calculated as the percentage of CFSE^lo^CD8^+^ T cells in each experimental condition (with *NK_te_* or *NK_control_*) divided by the percentage of CFSE^lo^CD8^+^ T cells observed in the absence of NK cells for each donor, multiplied by 100.

### Statistical Methods

Differences between data sets were analyzed with the two-sided Student’s t-test, or with Wilcoxon or Mann-Whitney tests; one-way ANOVA (when more than two experimental groups were compared) or two-way ANOVA (when two variables were analyzed), with Dunnett´s, Tukey’s or Sidak´s multiple *post hoc* tests; and the correlation between two variables was analyzed with Pearson´s coefficient using GraphPad Prism Software.

## Results

### NK Cell Infiltration in ccRCC Patients Is Associated With Decreased Survival

**Figure 1:**
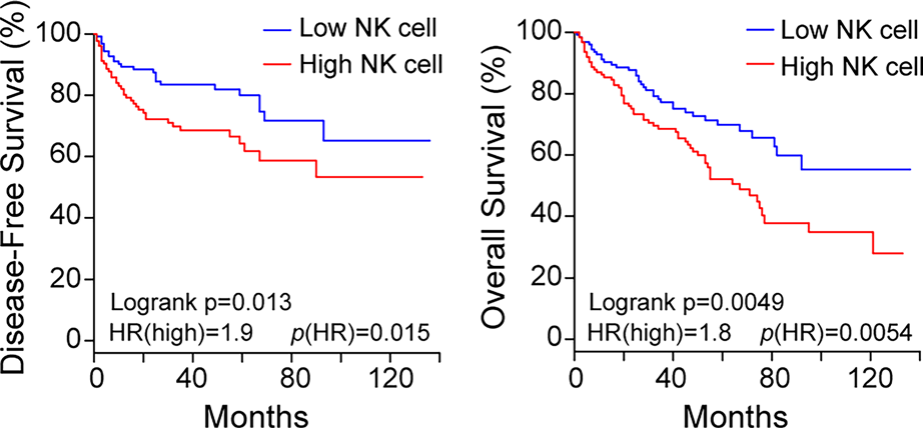
The presence of a high NK cell infiltration in ccRCC is associated with decreased survival. Kaplan-Meier curves for patients with KIRC (n = 516) from the TCGA database. Disease-free survival (left) and overall survival (right) of patients with high (red line) and low (blue line) NK cell infiltration was established according to the abundance of a gene signature that includes 10 NK cell-associated genes (NCR1, XCL1, XCL2, NCAM1, NCR3, IL18RAP, KIR2DL4, KLRC3, KLRD1 and NCR2, as explained in Materials and Methods). Hazard ratio (HR) for the high NK infiltration and logrank *p* values are shown in the lower left of each Kaplan-Meier plot. Top and bottom quartiles are shown.

To investigate the impact of TINK on patient survival, first we analyzed TCGA ccRCC datasets using the GEPIA2 tool. We used a NK cell gene signature as surrogate marker of NK cell infiltration. Kaplan-Meier analysis revealed that a higher NK cell infiltration (NK^high^) is associated with a poor outcome, both in terms of disease-free survival and overall survival (**Figure 1**). These results suggest that TINK in ccRCC might have a paradoxical deleterious impact on tumor growth control.

### PD-L1 Is Up-Regulated in NK Cells From ccRCC Patients

**Figure 2:**
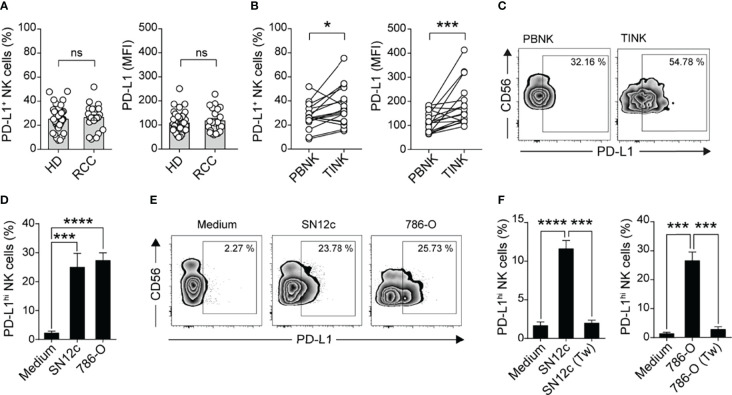
TINK cells from patients with ccRCC exhibit an accumulation of PD-L1^+^ cells and increased expression of PD-L1. **(A)** Frequency of PD-L1^+^ NK cells (left) and expression of PD-L1 (right) on PBNK from healthy donors (HD, n=41) and ccRCC patients (n=22), assessed by flow cytometry. **(B)** Frequency of PD-L1^+^ NK cells (left) and MFI of PD-L1 (right) on PBNK and TINK from matched ccRCC patients, assessed by flow cytometry (n=17). **(C)** Representative zebra plots of CD56 vs PD-L1 on PBNK and TINK from a representative ccRCC patient. **(D)** Frequency of PD-L1^hi^ NK cells after culture of PBMC from HD in the absence (Medium) or in the presence of SN12c or 786-O RCC cell lines for 48 h, assessed by flow cytometry (n=12). **(E)** Representative zebra plots. **(F)** Frequency of PD-L1^hi^ NK cells after culture of PBMC from HD in the absence (Medium) or in the presence of SN12c or 786-O RCC cell lines in contact or separated by transwells (Tw) for 48 h, assessed by flow cytometry (n=7). Two-sided Student´s t test **(A, B)**; one-way ANOVA with Dunnett´s *post hoc* test **(D, F)**. Data represent mean ± SEM **(D, F)**. ns, non-significant; *p<0.05, ***p<0.001, ****p<0.0001.

Previously, we observed that tumor-bearing mice exhibit an increased frequency of NK cells that express the inhibitory molecule PD-L1, and that they can limit tumor antigen-specific CD8 T cell priming (30). As the expression of PD-L1 on human NK cells remains underexplored, we sought to investigate if human NK cells from ccRCC patients also contain an increased frequency of cells that express PD-L1. We analyzed the frequency of TINK cells within CD45^+^ cells in fresh ccRCC samples by flow cytometry and observed that ccRCC tumors are infiltrated by NK cells, with frequencies ranging from 3.21% to 54.61%. We next assessed the expression of PD-L1 on NK cells from peripheral blood (PBNK) and TINK from these ccRCC patients. The frequency of PD-L1^+^ NK cells and the intensity of PD-L1 expression was similar among PBNK from healthy donors (HD) and ccRCC patients (**Figure 2A**). However, the frequency of TINK that express PD-L1 and the intensity of PD-L1 expression on these TINK was significantly higher than in PBNK of the same patient (**Figures 2B, C** **)**. PD-L1 up-regulation could also be induced *in vitro* on PBNK from HD upon coculture with the NK cell-sensitive RCC cell lines SN12c and 786-O (**Figures 2D, E** **)**. Cell contact with tumor cells was essential for the induction of PD-L1 expression since the separation of PBMC from SN12c and from 786-O cells by transwells prevented PD-L1 up-regulation (**Figure 2F**). Therefore, direct interaction with RCC tumor cells promotes the up-regulation of PD-L1 on NK cells that resemble NK cells detected in the tumor niche.

### Tumor Recognition and PBMC-Derived Factors Induce PD-L1 on NK Cells

**Figure 3:**
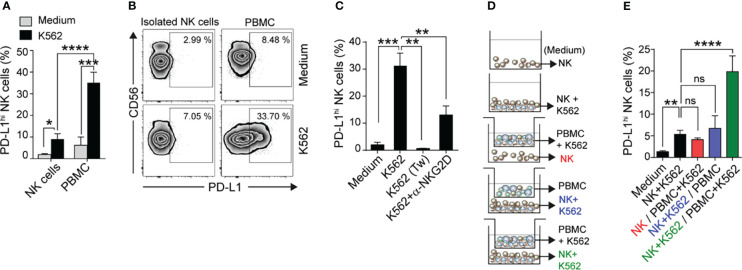
Emergence of PD-L1^hi^ NK cells requires contact with tumor cells and PBMC-derived soluble factors. **(A)** Frequency of PD-L1^hi^ NK cells after culture of isolated NK cells or PBMC in the absence (Medium) or in the presence of K562 cells for 48 h, assessed by flow cytometry (n=7-11). **(B)** Representative zebra plots. **(C)** Frequency of PD-L1^hi^ NK cells after culture of PBMC in the absence (Medium) or in the presence of K562 cells, in contact or separated by a Tw, or in the presence of anti-NKG2D blocking mAb for 48 h, assessed by flow cytometry (n=4). **(D)** Experimental design used to assess the requirements for the induction of PD-L1^hi^ NK cells. Isolated NK cells, PBMC and K562 cells were cultured separated by Tw as indicated in the figure. **(E)** Frequency of PD-L1^hi^ NK cells after culture of isolated NK cells in the absence (Medium) or in the presence of K562 cells or PBMC together or separated by Tw, assessed by flow cytometry (n=3). Data represent mean ± SEM. Two-way ANOVA with Sidak´s multiple comparison **(A)** and one-way ANOVA with Dunnett´s **(C)** and Tukey´s **(E)** posthoc. ns, non-significant; *p<0.05, **p<0.01, ***p<0.001, ****p<0.0001.

To investigate the mechanisms underlying PD-L1 up-regulation on NK cells, we cultured isolated NK cells or whole PBMC from HD with the HLA-null cell line K562 (to eventually prevent effects mediated by differences in KIR receptors) for 48 h and evaluated the frequency of PD-L1^hi^ NK cells. We observed a higher frequency of PD-L1^hi^ NK cells when NK cells were exposed to K562 tumor cells in the presence of other cells present in PBMC, suggesting that PBMC can provide costimulatory signals that result in a more efficient PD-L1 up-regulation (**Figures 3A, B** **)**. Nonetheless, cell contact with tumor cells was essential for the induction of PD-L1 (**Figure 3C** **)**. Furthermore, the effect was partially dependent on NKG2D engagement on NK cells, as NKG2D blockade interfered with the up-regulation of PD-L1 (**Figure 3C**). To investigate the nature of the PBMC-derived factors that contributed to PD-L1 up-regulation on NK cells, we cultured NK cells or NK cells in contact with K562 cells (bottom compartment) separated by transwells from PBMC or K562 cells and PBMC (top compartment), as depicted in **Figure 3D**, and assessed the frequency of PD-L1^hi^ NK cells in the bottom compartment (**Figure 3E**). Whereas NK cells that only sensed soluble factors derived from PBMC and K562 cells exhibited a marginal up-regulation of PD-L1, NK cells in contact with K562 cells that received PBMC-derived soluble factors exhibited a minor but significant PD-L1 up-regulation. Instead, NK cells that experienced direct cell contact with K562 cells and sensed K562-experienced PBMC-derived soluble factors exhibited a marked up-regulation of PD-L1, resulting in an increased frequency of PD-L1^hi^ NK cells (**Figure 3E**). Thus, contact between NK cells and tumor cells in cooperation with soluble factors derived from K562-experienced PBMC strongly induce PD-L1 expression on NK cells.

### PBMC-Derived IL-18 Enhance PD-L1 Expression on Tumor-Experienced NK Cells

**Figure 4:**
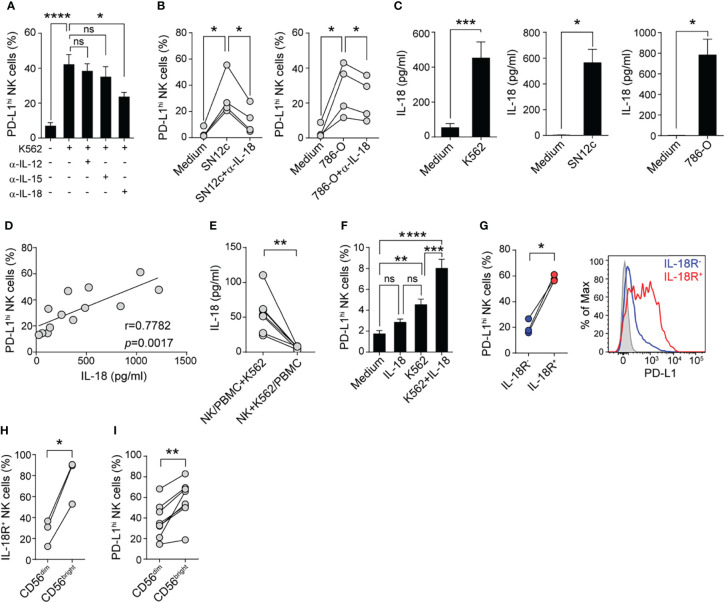
IL-18 produced during tumor-stimulation of PBMC enhances PD-L1 expression on tumor-experienced NK cells. **(A)** Frequency of PD-L1^hi^ NK cells after culture of PBMC in the absence (Medium) or in the presence of K562 cells and different cytokine-neutralizing mAb for 48 h, assessed by flow cytometry (n=5). **(B)** Frequency of PD-L1^hi^ NK cells after culture of PBMC in the absence (Medium) or in the presence of SN12c cells (left) or 786-O cells (right) and in the absence or in the presence of an anti-IL-18-neutralizing mAb for 48 h, assessed by flow cytometry (n=4). **(C)**, IL-18 concentration in supernatants of PBMC cultured in the absence (Medium) or presence of K562 cells (n=13), SN12c cells (n=4) or 786-O cells (n=4), assessed by ELISA. **(D)** Correlation between the concentration of IL-18 detected in the culture supernatants of PBMC stimulated with K562 cells and the frequency of PD-L1^hi^ NK cells (n=13). **(E)** IL-18 concentration in supernatants of PBMC cultured in contact with K562 cells and separated from isolated NK cells by Tw (left) or PBMC separated from isolated NK cells and K562 cells by Tw (right), assessed by ELISA (n=7). **(F)** Frequency of PD-L1^hi^ NK cells after culture of isolated NK cells in the absence (Medium) or in the presence of IL-18, K562 cells or K562 cells and IL-18 (K562+IL-18) for 48 h, assessed by flow cytometry (n=10). **(G)** Frequency of PD-L1^hi^ cells detected in IL-18Rα^+^ (red) and IL-18Rα^-^ NK cells (blue) after stimulation of PBMC with K562 cells for 48 h, assessed by flow cytometry (n=3). A representative histogram is shown. **(H)** Frequency of IL-18Rα^+^ cells among CD56^dim^ and CD56^bright^ NK cells after culture of PBMC in the presence of K562 cells for 48 h (n=3). **(I)** Frequency of PD-L1^hi^ cells among CD56^dim^ and CD56^bright^ NK cells after culture of PBMC in the presence of K562 cells for 48 h (n=8). Data represent mean ± SEM. One-way ANOVA with Dunnett´s **(A, B)** and Tukey´s **(F)** *post hoc* test, two-sided Student´s t test **(C, E, G–I)** and Pearson´s correlation **(D)**. ns, non-significant; *p<0.05, **p<0.01, ***p<0.001, ****p<0.0001.

To unravel possible PBMC-derived soluble mediators involved in PD-L1 up-regulation, we neutralized different cytokines known to activate NK cells (IL-12, IL-15, or IL-18) with specific mAb during PBMC and K562 cocultures and after 48 h NK cells were evaluated for PD-L1 expression. IL-18 neutralization, but not IL-12 or IL-15 neutralization, interfered with PD-L1 up-regulation on NK cells (**Figure 4A**), suggesting that IL-18 produced during tumor-stimulation of PBMC contributes to enhance PD-L1 expression on NK cells. Similarly, IL-18 neutralization interfered with PD-L1 up-regulation on NK cells present in PBMC from HD cultured with SN12c cells or with 786-O cells (**Figure 4B**). Accordingly, PBMC stimulation with K562 cells, SN12c cells or 786-O cells resulted in a significantly increased IL-18 production (**Figure 4C**) and the concentration of IL-18 in the supernatant of PBMC stimulated with K562 positively correlated with the frequency of PD-L1^hi^ NK cells (**Figure 4D**). K562-driven IL-18 production by PBMC required cell-to-cell contact as it was prevented when PBMC were separated from K562 and NK cells using transwells (**Figure 4E**). Moreover, conditioned media from K562 cells cultured with NK cells barely induced IL-18 secretion by PBMCs (*not shown*). In addition, supplementation of NK cell cultures with IL-18 during stimulation with K562 cells significantly increased the expression of PD-L1 on NK cells (**Figure 4F**). Moreover, PD-L1 expression was preferentially induced on IL-18Rα^+^ NK cells (**Figure 4G**). Next, we analyzed IL-18Rα and PD-L1 expression in CD56^dim^ and CD56^bright^ NK cell present in PBMC stimulated with K562 cells. A higher frequency of IL-18Rα^+^ cells was observed among CD56^bright^ NK cells (**Figure 4H**), and the frequency of PD-L1^hi^ cells was higher in CD56^bright^ NK cells (**Figure 4I**). These results suggest that PD-L1 becomes preferentially expressed on IL-18 responsive (IL-18Rα^+^) NK cells. Altogether, these results identify IL-18 as a critical cytokine that contributes to PD-L1 up-regulation on tumor-experienced NK cells.

### Monocyte-Derived IL-18 Accounts for the Accumulation of PD-L1^hi^ NK Cells

**Figure 5:**
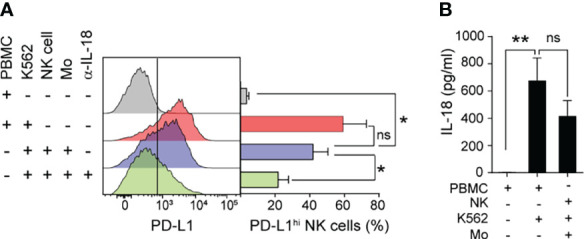
Monocyte-derived IL-18 induces PD-L1 expression on tumor-experienced NK cells. **(A)** Frequency of PD-L1^hi^ NK cells after culture of PBMC in the absence or in the presence of K562, isolated NK cells in the presence of K562 cells and/or autologous monocytes (Mo), in the absence or in the presence of an anti-IL-18 neutralizing mAb for 48 h, assessed by flow cytometry. **(B)** concentration of IL-18 in supernatants of the cultures, assessed by ELISA. Data represent mean ± SEM, (n=4). One-way ANOVA with Dunnett´s *post hoc* test. ns, non-significant; *p<0.05, **p<0.01.

As monocytes constitute the main cell population within PBMC that can produce IL-18, we analyzed the frequency of PD-L1^hi^ NK cells when isolated NK cells were stimulated with K562 cells in the presence of autologous monocytes. Monocytes significantly increased the expression of PD-L1 on NK cells, to the level observed when whole PBMC were cultured with K562 cells, and this effect was partially reverted by IL-18 blockade (**Figure 5A**). Accordingly, monocytes cultured with NK cells and K562 cells (or RCC cell lines, *not shown*) produced similar amounts of IL-18 than PBMC cultured with tumor cells (**Figure 5B**), and separation of monocytes from NK cells and tumor cells by transwells abrogated IL-18 secretion (*not shown*). These results support the idea that monocytes sense the presence of tumor cells through cell contact and respond secreting IL-18, which in turn enhance PD-L1 expression on tumor-stimulated NK cells.

### Features of PD-L1^hi^ NK Cells

**Figure 6:**
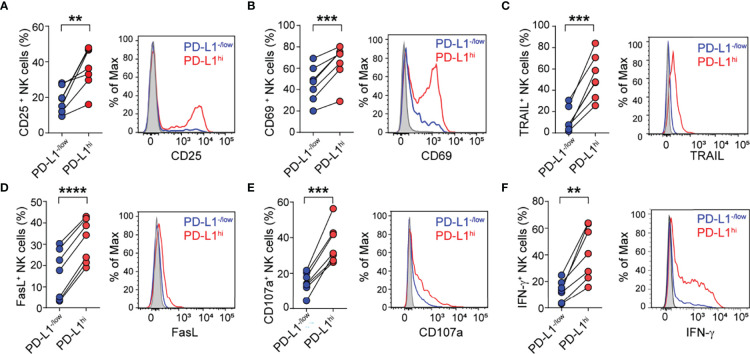
PD-L1^hi^ NK cells exhibit an activated phenotype and express higher cytotoxic mediators. Frequency of CD25^+^ cells **(A)**, CD69^+^ cells **(B)** TRAIL^+^ cells **(C)**, FasL^+^ cells **(D)**, CD107a^+^ cells **(E)** and IFN-γ + cells **(F)** detected in PD-L1^hi^ (red) and PD-L1^-/low^ NK cells (blue) after stimulation of PBMC with K562 cells for 48 h, assessed by flow cytometry (n=7). A representative histogram for each marker is shown. Two-sided Student´s t test **p<0.01, ***p<0.001, ****p<0.0001.

To characterize the phenotype and function of PD-L1^hi^ NK cells, PBMC from HD were cultured with K562 cells for 48 h, and the phenotype and effector function of PD-L1^hi^ versus PD-L1^-/low^ NK cells was analyzed. PD-L1^hi^ NK cells exhibited a higher frequency of CD25^+^ (**Figure 6A**), CD69^+^ (**Figure 6B**), TRAIL^+^ (**Figure 6C**) and FasL^+^ cells (**Figure 6D**) compared to PD-L1^-/low^ NK cells, suggesting that PD-L1 is preferentially expressed on NK cells that have undergone activation and acquired tissue homing and cytotoxicity effector molecules. In addition, PD-L1^hi^ NK cells also exhibited higher degranulation (CD107a expression) and IFN-γ production capacity than PD-L1^-/low^ NK cells (**Figures 6** **E, F**), confirming that PD-L1^hi^ NK cells, in opposition to PD-L1^-/low^ NK cells, display an activated phenotype and are highly functional.

### Tumor-Experienced NK Cells Limit CD8^+^ T Cell Proliferation Through PD-L1

**Figure 7:**
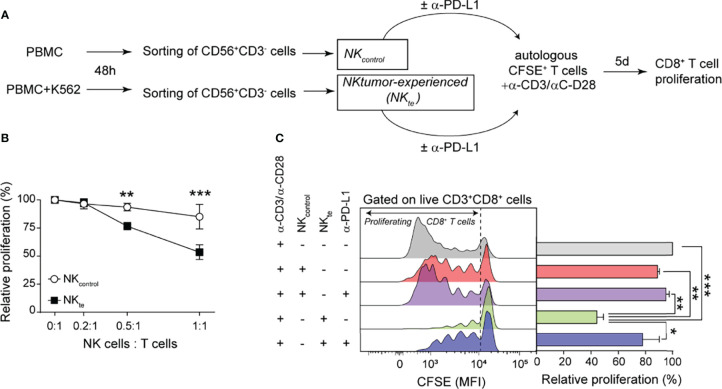
Tumor-experienced NK cells inhibit CD8^+^ T cell proliferation through PD-L1. **(A)** Experimental design used to generate control NK cells (*NK_control_*) and tumor-experienced NK cells enriched in PD-L1^hi^ cells (*NK_te_*) to investigate their effect on T cell proliferation. **(B)** Relative proliferation of CD8^+^ T cells analyzed by CFSE dilution after stimulation with anti-CD3/anti-CD28 mAb and cultured with increasing numbers of autologous *NK_control_* or *NK_te_*. **(C)** Relative proliferation of CD8^+^ T cells analyzed by CFSE dilution after stimulation with anti-CD3/anti-CD28 mAb in the absence or in the presence of autologous *NK_control_* or *NK_te_* in the absence or in the presence of an anti-PD-L1 blocking mAb. Representative histograms showing CFSE dilution in live CD3^+^CD8^+^CD56^-^ cells are shown (n=3). Data represent mean ± SEM. Two-way ANOVA with Sidak´s *post hoc* test **(B)** and one-way ANOVA and Tukey´s *post hoc* test **(C)** *p<0.05, **p<0.01, *** *p*<0.001.

As the PD-1/PD-L1 axis constitutes a major co-inhibitory pathway in T cells, we wondered whether PD-L1^hi^ NK cells contribute to constrain CD8^+^ T cell proliferation. Therefore, we performed T cell proliferation assays in the presence of tumor-experienced autologous NK cells enriched in PD-L1^hi^ NK cells (*NK_te_*) or naïve NK cells (*NK_control_*), following the experimental design depicted in **Figure 7A**. We stimulated CFSE-labeled T cells with anti-CD3 and anti-CD28 mAb in the absence or in the presence of growing numbers of *NK_control_* or *NK_te_* and after 5 d we assessed the proliferation of CD8^+^ T cells (CFSE dilution). The extent of CD8^+^ T cell proliferation was minimally affected by NK_control_ cells but decreased as the proportion of *NK_te_* cells in the cultures increased (**Figure 7B**). Also, PD-L1 blockade restored CD8^+^ T cell proliferation, indicating that the effect was PD-L1-dependent (**Figure 7C**). These results indicate that tumor-experienced NK cells can limit CD8^+^ T cell expansion through PD-L1 engagement.

## Discussion

NK cells display anti-tumor activity and a higher natural cytotoxic activity in peripheral blood has been associated with lower tumor incidence (4). However, a regulatory role for NK cells has been described during viral infection (14–17), transplantation (18) and autoimmunity (19). We recently demonstrated that in mice NK cells also can acquire a regulatory phenotype characterized by a tumor-induced up-regulation of PD-L1 (30). Therefore, although a dual role for NK cells is emerging, the impact of this dual role in tumor progression remains ill-defined. To get a first insight into this subject, we analyzed publicly available RNAseq and survival data from ccRCC patients deposited at TCGA. As an estimate of NK cell infiltration, we used a gene signature for NK cells based on previously published NK cell signatures (42–45) that includes 10 NK cell-associated genes not expressed or weakly expressed on CD8^+^ T cells (NCR1, XCL1, XCL2, NCAM1, NCR3, IL18RAP, KIR2DL4, KLRC3, KLRD1 and NCR2). Patients with a high NK cell infiltration (assessed as presence of a high expression of NK cell signature genes) exhibited a worse outcome than the group of patients with a low NK cell infiltration, which is in accordance with previous reports (46). A possibility that explains this finding is that TINK in ccRCC might facilitate tumor growth. However, it is also possible that tumors that developed more proficient strategies to subvert NK cell effector function exhibit higher tumor growth, resulting in lower disease-free and overall survival. TINK often are dysfunctional and display phenotypic alterations compared to NK cells from blood or from peritumoral tissue in different cancer types (8, 9, 11), including RCC (10, 12, 47). Using a mouse model, we previously demonstrated that TINK express PD-L1 and inhibit anti-tumor T cell responses. In the present work, we observed that TINK from human ccRCC patients exhibit an increased frequency of PD-L1^+^ cells and display an enhanced expression of PD-L1 compared to PBNK. Moreover, two RCC cell lines, one corresponding to a ccRCC (786-O), promoted the appearance of PD-L1^hi^ NK cells in PBMC from healthy donors. A large meta-analysis showed that PD-L1 expression in RCC tumor cells is associated with poorer overall survival (48), while PD-L1 expression on tumor-infiltrating immune cells is associated with poor prognosis in ccRCC patients (49). Therefore, we aimed at dissecting the mechanisms underlying the emergence of PD-L1^hi^ NK cells. We demonstrated that PBMC-derived factors, more precisely IL-18 produced by monocytes, plus NK cell-tumor cell contact that involves NKG2D were necessary to induce the up-regulation of PD-L1 on NK cells. Because IL-18 neutralization only partially blocked PD-L1 up-regulation on NK cells, suggests the participation of other soluble factors. Nonetheless, the preferential expression of PD-L1 on IL-18-responsive (IL-18Rα^+^) NK cells supports our conclusions. IFN-γ is a positive regulator for PD-L1 expression in multiple cell types (32, 50). However, PD-L1 expression on NK cells remained unchanged when PBMC were stimulated with K562 cells in the presence of an anti-IFN-γ receptor blocking mAb or an anti-IFN-γ neutralizing mAb, or when isolated NK cells were stimulated with IFN-γ (*not shown*). Instead, our results indicate that IL-18 produced by monocytes represents the main factor that enhanced PD-L1 expression in tumor-stimulated NK cells. These findings might indicate that PD-L1^hi^ NK cells may even appear when NK cell and T cell-derived IFN-γ production is waned because of different tumor-escape mechanisms. IL-18 is a cytokine with pro-inflammatory properties, involved in stimulation of NK cells and Th1-biased immune responses that are crucial for anti-tumor immunity (51). However, IL-18 also has a dual role as the presence of high amounts of IL-18 in serum of patients with tumors of different etiology, including RCC, is associated with disease progression and aggressiveness, and correlates with advanced tumor stage, worsened outcome and shorter overall survival (52–54). In this scenario, IL-18-driven emergence of PD-L1^hi^ NK cells might be part of the tumor-promoting effects of this cytokine.

Although PD-L1 expressed on tumor cells plays a relevant role in attenuating anti-tumor immunity, different studies using preclinical mouse models showed that PD-L1 expression on non-tumor cells plays non-overlapping roles in the suppression of anti-tumor T cell responses and contributes to tumor escape (36, 37). Moreover, the efficacy of anti-PD-L1 therapy relies partially on PD-L1 expression on non-tumor cells (38, 39, 55). Accordingly, our results showing that ccRCC-infiltrating NK cells display an increased frequency of PD-L1^hi^ NK cells, and that tumor-experienced NK cells enriched in PD-L1^hi^ NK cells can inhibit CD8^+^ T cell proliferation through PD-L1, suggest that NK cells also might be targeted during immune checkpoint treatment of cancer patients with anti-PD-1/PD-L1 mAb. Accordingly, the presence of PD-L1^+^ NK cells in blood of acute myeloid leukemia patients was recently reported, and treatment with atezolizumab, a humanized anti-PD-L1 mAb, increased the effector functions of PD-L1^+^ NK cells (56). In addition, as PD-1 can be expressed on a subset of NK cells, as observed in CMV-infected and in cancer patients (57), it is also possible that PD-L1^hi^ NK cells might display a regulatory activity on PD-1^+^ NK cells, either *via cis* or *trans* interaction.

Our results showing that *in vitro* generated PD-L1^hi^ NK cells display enhanced effector functions and an activated phenotype might contrast with our initial observation that high TINK infiltration correlates with a worsened outcome. However, as NK cell activation occurs early after encounter with tumors, and PD-L1 up-regulation is observed later, peaking at 48 h, it is conceivable that PD-L1 becomes up-regulated preferentially on activated NK cells as part of a homeostatic program. Also, analysis of TINK from sarcoma and breast tumor patients, demonstrated co-expression of multiple immune checkpoints including PD-L1, CD73, TIM-3, LAG-3, VISTA and PD-1 (58). These data indicate that chronically stimulated TINK acquire features of exhausted cells, in opposition to NK cells stimulated *in vitro* for only 48 h as we used in this work. Moreover, the immunosuppressive tumor microenvironment also operates on NK cells, rendering them exhausted and dysfunctional.

CD8^+^ T cells are critical for tumor elimination and the presence of tumor-infiltrating CD8^+^ T cells has a positive prognostic value in patients with different cancer types (59). Moreover, in ccRCC patients a higher proliferative activity of tumor-infiltrating CD8^+^ T cells is associated with longer survival (60). Our results showing that tumor-experienced NK cells were able to inhibit CD8^+^ T cell proliferation could explain why a high infiltration of NK cells is associated with reduced survival.

In summary, tumor recognition, in cooperation with monocyte-derived IL-18, induce the expression of PD-L1 on NK cells, resulting in an enrichment in PD-L1^hi^ NK cells that in turn limit CD8^+^ T cell proliferation in a PD-L1-dependent manner. Therefore, the clinical response observed in several tumor types, including ccRCC, after therapeutic targeting the PD-1/PD-L1 axis using specific checkpoint inhibitors might be partially dependent on PD-L1 blockade on TINK.

## Data Availability Statement

The raw data supporting the conclusions of this article will be made available by the authors, without undue reservation.

## Ethics Statement

The studies involving human participants were reviewed and approved by Institutional Ethics Committee of IBYME (protocol CE003-03/2014, date of approval: March 20, 2014). The patients/participants provided their written informed consent to participate in this study.

## Author Contributions

JS participated in the design of the study, performed and analyzed the experiments, contributed to the preparation of the figures, and corrected the manuscript. FS, SN, XR, AZ, MG, AT, AF, MR, MS, NT, CD, and NZ assisted in performing experiments and analyzing results. CA, GV, HR, LR, AR, and NR provided RCC samples. NZ provided advice and contributed to manuscript writing. MF designed the study, analyzed experiments, prepared figures and wrote the manuscript. All authors reviewed the results and approved the final version of the manuscript. All authors contributed to the article and approved the submitted version.

## Funding

This work was supported by grants from the National Agency for Promotion of Science and Technology from Argentina (ANPCYT) and the National Research Council of Argentina (CONICET) and the Trust in Science Program from GlaxoSmithKline (GSK), granted to MF and NZ. The funder was not involved in the study design, collection, analysis, interpretation of data, the writing of this article or the decision to submit it for publication. We also thank Fundación Williams, Fundación Cherny and Fundación René Barón for providing additional support.

## Conflict of Interest

The authors declare that the research was conducted in the absence of any commercial or financial relationships that could be construed as a potential conflict of interest.

## Publisher’s Note

All claims expressed in this article are solely those of the authors and do not necessarily represent those of their affiliated organizations, or those of the publisher, the editors and the reviewers. Any product that may be evaluated in this article, or claim that may be made by its manufacturer, is not guaranteed or endorsed by the publisher.
