# Supplementary figures and images for: Tumor-Experienced Human NK Cells Express High Levels of PD-L1 and Inhibit CD8+ T Cell Proliferation
Source: Front Immunol. 2021 Sep 20;12:745939. doi: 10.3389/fimmu.2021.745939 (PMC8488336; doi:10.3389/fimmu.2021.745939)

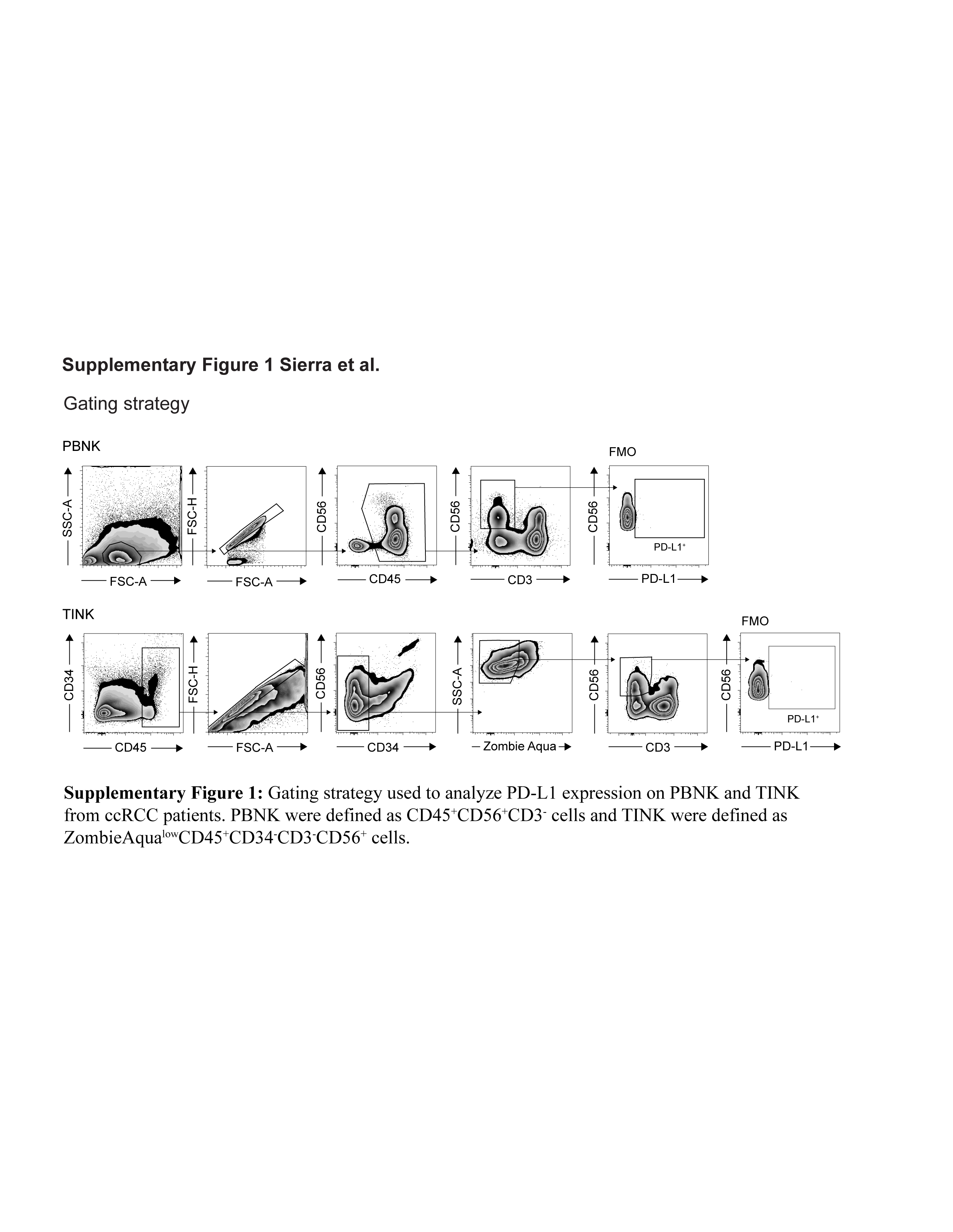

Supplement: Supplementary file 1 [file Image_1.tif]

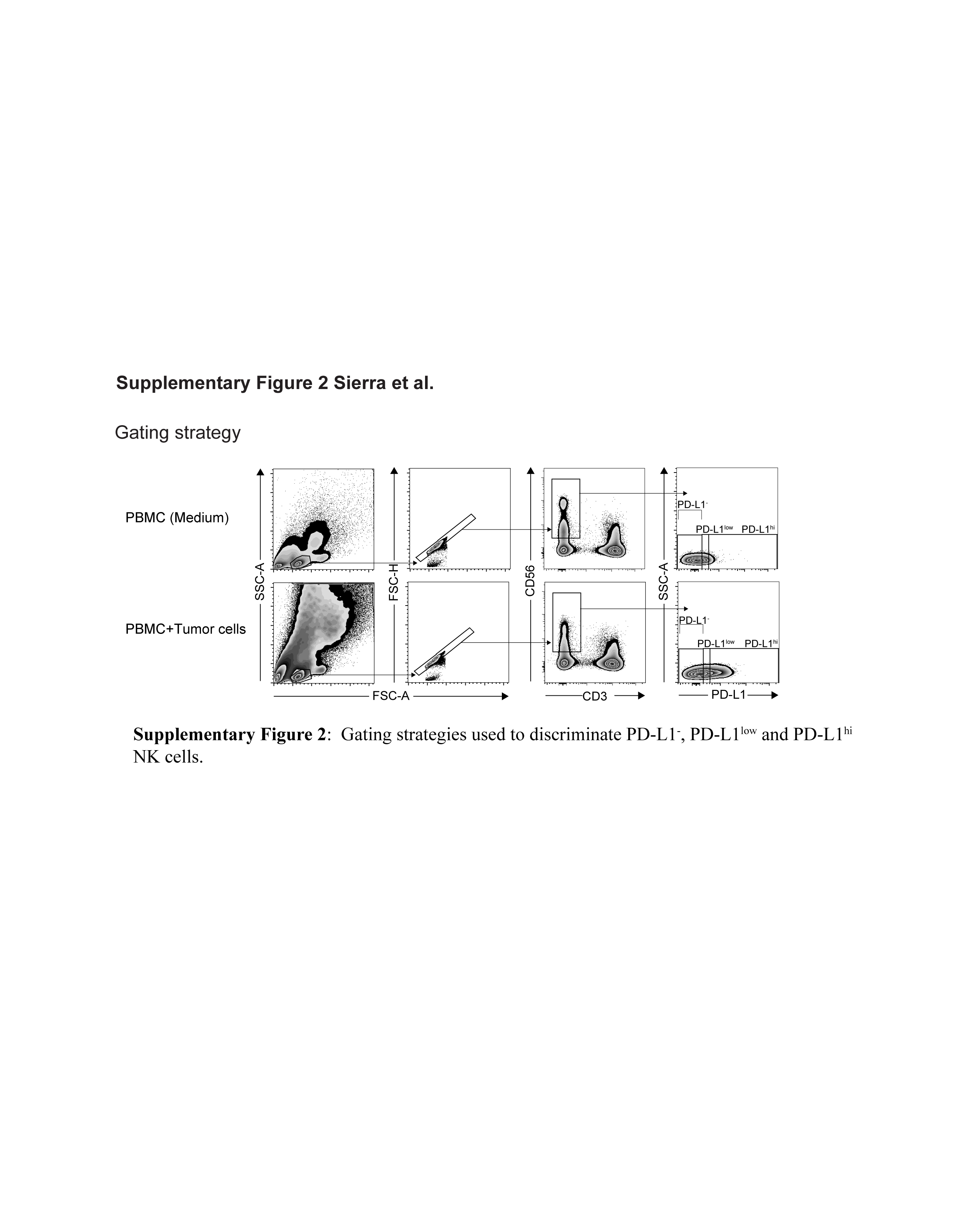

Supplement: Supplementary file 2 [file Image_2.tif]

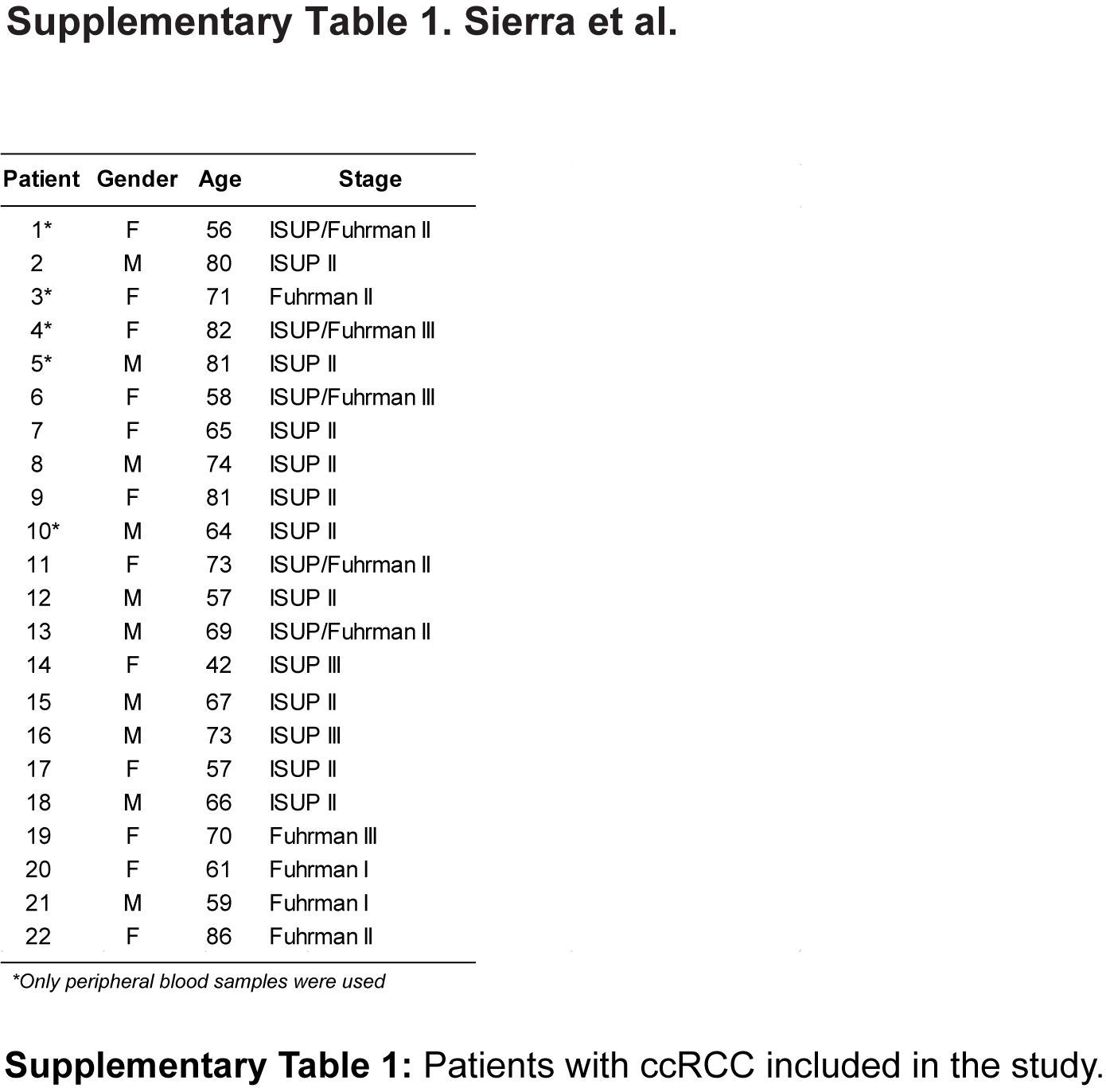

Supplement: Supplementary file 3 [file Image_3.tif]
